# Risk Factors for Atrial Arrhythmias in Adults With Ebstein Anomaly

**DOI:** 10.1016/j.jacadv.2022.100058

**Published:** 2022-08-26

**Authors:** Irene Martin de Miguel, William R. Miranda, Malini Madhavan, Heidi M. Connolly, Joseph A. Dearani, Alexander C. Egbe

**Affiliations:** a Department of Cardiovascular Medicine, Mayo Clinic, Rochester, Minnesota, USA; b Department of Cardiovascular Surgery, Mayo Clinic, Rochester, Minnesota, USA

**Keywords:** atrial arrhythmia, atrial fibrillation, atrial flutter, Ebstein anomaly, left atrial volume index, right atrial reservoir strain

## Abstract

**Background:**

Atrial arrhythmias (AA) are common in Ebstein anomaly (EA), but risk factors associated with AA are not well understood.

**Objectives:**

The purpose of this study was to determine the prevalence and risk factors for AA at baseline, incidence, and risk factors for AA during follow-up.

**Methods:**

Adults with EA receiving care at Mayo Clinic, MN, between 2003 and 2020 were included. AA was defined as atrial fibrillation (AF) or atrial flutter/tachycardia (AFL). Clinical, echocardiographic, rhythm, surgical data were collected.

**Results:**

Of 682 patients (aged 36 [24-49] years), 235 (34%) had AA at baseline (126 [18%] AF and 144 [21%] AFL), and the risk factors for AA were age, left and right atrial volume indexes, and reservoir strain. Among 447 patients without AA, 10-year cumulative incidence of AF and AFL was 16% and 22%, respectively. The risk factors for incident AF were older age and right atrial reservoir strain. The risk factors for incident AFL were atrial septal defect, left atrial volume index, and male sex. Among patients with baseline AA, 129 (40%) had recurrent episodes (AF 63 [20%], AFL 78 [24%]). The 5-year recurrence rate of AA was 34%, without significant difference for AF vs AFL (46% vs 27%, *P* = 0.081). Older age and right atrial reservoir strain were associated with recurrent AF.

**Conclusions:**

Patients with EA are at risk for incident and recurrent AA. AF was almost as common as AFL despite relatively young ages. Echocardiographic indexes of atrial function can identify at-risk patients, hence be used to improve risk stratification and guide therapy.

Ebstein anomaly (EA) is characterized by tricuspid valve dysplasia and right ventricular myopathy, and, as a result, the natural history is influenced by the severity of tricuspid regurgitation and right heart dysfunction.^1^^,^^2^ Tricuspid valve surgery (repair or replacement) is effective in preventing progressive right heart volume overload and remodeling, especially when performed before the onset of irreversible cardiac dysfunction.^3^^,^^4^

Atrial arrhythmias (AA) are common in adults with EA.^5^, ^6^, ^7^ The etiology of AA is largely attributed to advanced cardiac remodeling from chronic right heart volume overload, and the onset of AA is associated with adverse outcomes.^8^, ^9^, ^10^ As a result, several treatment strategies such as antiarrhythmic drugs (AADs), catheter ablation, and antiarrhythmic surgery have been used for the prevention and treatment of AA in this population.^11^, ^12^, ^13^ Data on the prevalence, risk factors, and treatment outcomes for AA in patients with EA are mainly derived from small studies or studies based on a mixed cohort of children and adults.^14^, ^15^, ^16^, ^17^, ^18^ Characterization of risk factors associated with AA using a large well-characterized cohort will provide the foundation for interventions to reduce the burden of arrhythmias and improve outcomes in this population. Our objectives were to determine: 1) the prevalence and risk factors associated with AA at the time of baseline assessment and; 2) the incidence and risk factors associated with new-onset and recurrent AA during follow-up in a cohort of adults with EA.

## Methods

### Study population

This is a retrospective cohort study of consecutive adults (aged ≥18 years) with EA receiving care at Mayo Clinic, Minnesota, USA, between January 1, 2003, and December 31, 2020. Patients were identified using an electronic search tool querying medical and surgical records. The Mayo Clinic Institutional Review Board approved this study and waived informed consent for patients that provided research authorization. Clinical, echocardiographic, electrocardiographic, and laboratory data as well as surgical and electrophysiology procedural reports were reviewed in all patients. The first clinical evaluation performed in the Adult Congenital Heart Disease Clinic on or after January 1, 2003, was considered as the baseline, and clinical indexes obtained within the 12 months from this assessment were used to define the baseline clinical characteristics of the cohort.

### Atrial arrhythmia

The diagnosis of AA was based on a manual review of 12-lead electrocardiograms, Holter monitors, rhythm strips, and device interrogation. AA was defined as atrial fibrillation (AF), atrial flutter (AFL), or atrial tachycardia. Given the difficulties in differentiating AFL and atrial tachycardia on a 12-lead electrocardiogram, AFL and atrial tachycardia were combined as a single diagnosis for the purpose of this study. Only arrhythmias lasting longer than 30 seconds were included.

In patients undergoing electrophysiology study, AF was defined as an AA with irregular atrial activation and cycle lengths below 200 ms, AFL as a macro-re-entrant AA characterized by a constant cycle length with a stable activation sequence that was entrainable, and focal atrial tachycardia as an AA originating from a focal source that was not entrainable.^19^

“AA” that were present at the time of, or before, baseline assessment were defined as “prevalent AA,” whereas AA occurring from baseline evaluation to the last follow-up were considered as “incident AA”; hence, time 0 for assessing incident AA was the baseline encounter. “Recurrent AA” were defined as ≥2 episodes of AA occurring anytime during follow-up in a patient with prior history of AA. All patients with prevalent or incident AA were potential candidates for developing recurrent AA. Antiarrhythmic therapies were defined as the use of AAD (Class I or III AAD), catheter ablation, or antiarrhythmic surgery (right atrial [RA] Maze/isthmus ablation and left atrial Maze).

### Echocardiographic assessment

Comprehensive transthoracic echocardiogram was performed according to contemporary guidelines with off-line image analyses and measurements performed by 2 experienced research sonographers (James Welper and Katrina Tollefsrud).^20^, ^21^, ^22^ The intra- and inter-observer agreement for this database has been previously described.^23^ RA and left atrial reservoir strain and right ventricular global longitudinal strain were calculated using speckle tracking strain imaging obtained with Vivid E9 and E95 (General Electric Co) with M5S and M5Sc-D transducers (1.5-4.6 MHz) at frame rate of 40 to 80 Hz. Three-beat cineloop clips were obtained from right ventricular-dedicated apical 4-chamber views. The ventricular septum was included for right ventricular global longitudinal strain calculation. Off-line analysis of the exported images was performed with TomTec (TomTec Imaging Systems). Adequate tracking by the software was verified and retraced if necessary. The rest of structure, function, and hemodynamic indexes were assessed with standard techniques.^20^, ^21^, ^22^

### Statistical analysis

Data were presented as count (percentage), mean ± SD, or median (IQR). Between-group comparisons of patients with vs without AA at baseline were performed using unpaired *t*-test and chi-squared test as appropriate.

The risk factors associated with prevalent AA, ie, AA that were present at the time of, or before baseline assessment, were assessed with multivariable logistic regression analysis using stepwise backward selection of covariates. The threshold for a covariate to remain in the model was set at *P* < 0.05. The covariates used in the multivariable logistic regression model were derived from univariable analysis of variables that were present at the time of baseline assessment, including age, sex, comorbidities, surgical history, echocardiographic indexes, and antiarrhythmic therapy. A threshold of *P* ≤ 0.10 in the univariable analysis was required to enter the multivariable model except for age and sex, which were always incorporated in the multivariable model irrespective of their *P* value in the univariable analysis.

The cumulative incidence of incident and recurrent AF and AFL was depicted using Kaplan-Meier curves. Incident AA was defined as any new-onset AA occurring in patients without a history of AA at the time of first clinical encounter. Recurrent AA was defined as any AA that occurred after: 1) the first clinical encounter in patients with a history of AA at baseline (time 0 = first clinical encounter); or 2) the first incident AA in patients without a history of AA at baseline but who developed an AA during follow-up (time 0 = time at the first incident AA). The risk factors associated with incident and recurrent AA were assessed with multivariable Cox regression analysis with stepwise backward selection using a similar method of covariate selection as in the logistic regression analysis. The proportionality of hazards of our models was confirmed via graphical inspection and Schoenfeld residuals. Owing to the large overall sample size and the large n of the different multivariable models, missing data were treated by deletion (complete case analysis). A *P* value <0.05 was considered statistically significant. All statistical analyses were performed with JMP software (version 14.1.0, SAS Institute Inc).

## Results

### Prevalent atrial arrhythmia

We identified 682 patients that met the study inclusion criteria. Table 1 shows baseline characteristics of the cohort. The median age was 36 (24-49) years, 41% were males, and 174 (26%) had prior repair of EA (tricuspid valve repair or replacement). Of the entire cohort, 126 (18%) had prior history of AF, and 144 (21%) had prior history of AFL. Overall, the prevalence of AA at the time of baseline assessment was 34% (n = 235). Compared with patients without prior history of AA, those with prevalent AA were older, more likely to be males, and had a higher prevalence of hypertension, renal dysfunction, prior tricuspid valve surgery, and cardiac implantable electronic devices.

**Table 1:** Baseline Characteristics

	N	All (N = 682)	N	AA (n = 235)	N	No AA (n = 447)	*P* Value
Demographics and comorbidities	682		235		447		
Male		279 (41.0)		114 (49.0)		165 (37.0)	0.004
Age at first visit, y		36 (24-49)		46 (32-56)		32 (22-45)	<0.001
Hypertension		104 (15.0)		47 (20.0)		57 (13.0)	0.014
Dyslipidemia		111 (16.0)		37 (16.0)		74 (17.0)	0.785
Diabetes mellitus		24 (4.0)		9 (4.0)		15 (3.0)	0.751
Obesity		126 (19.0)		46 (20.0)		80 (18.0)	0.593
Coronary artery disease		26 (4.0)		12 (5.0)		14 (3.0)	0.210
Previous stroke		38 (6.0)		16 (7.0)		22 (5.0)	0.314
Creatinine, mg/dL		0.98 ± 0.24		1.0 ± 0.24		0.97 ± 0.24	0.037
Associated congenital defects	682		235		447		
Atrial septal defect		353 (52.0)		133 (57.0)		220 (49.0)	0.067
Ventricular septal defect		40 (6.0)		18 (8.0)		22 (5.0)	0.156
Pulmonary stenosis		21 (3.0)		9 (4.0)		12 (3.0)	0.418
Prior cardiac procedures	682		235		447		
Cardiac surgery		174 (26.0)		79 (34.0)		95 (21.0)	<0.001
Tricuspid valve repair		114 (17.0)		55 (23.0)		59 (13.0)	<0.001
Tricuspid valve replacement		60 (9.0)		24 (10.0)		36 (8.0)	0.349
Bioprosthesis		58 (9.0)		23 (10.0)		35 (8.0)	0.389
Mechanical prosthesis		2 (0.3)		1 (0.4)		1 (0.2)	0.652
CIED		57 (8.0)		35 (15.0)		22 (5.0)	<0.001
Echocardiography							
RA volume index, mL/m^2^	609	56 (42-81)	209	66 (47-115)	400	52 (42-70)	<0.001
RA reservoir strain^a^, %	604	29 ± 14	206	22 ± 12	398	33 ± 13	<0.001
RA pressure, mm Hg	654	5 (5-10)	223	10 (5-15)	431	5 (5-10)	<0.001
≥Moderate tricuspid regurgitation	673	495 (74.0)	231	158 (68.0)	442	337 (76.0)	0.030
TR velocity, m/s	584	2.5 ± 0.9	179	2.4 ± 0.4	405	2.5 ± 1.1	0.108
RV end-diastolic area, cm^2^	610	45 ± 17	212	48 ± 18	398	43 ± 16	0.001
RV end-systolic area, cm^2^	610	31 ± 14	212	34 ± 15	398	30 ± 13	<0.001
RV global strain^a^, %	611	18 ± 6	212	16 ± 5	399	19 ± 6	<0.001
TAPSE, mm	614	22 ± 11	214	19 ± 10	400	24 ± 11	<0.001
RV systolic pressure, mm Hg	576	33 ± 8	177	33 ± 8	399	33 ± 7	0.283
LA volume index, mL/m^2^	638	25 ± 12	218	30 ± 15	420	22 ± 8	<0.001
LA reservoir strain^a^, %	607	32 ± 12	207	28 ± 12	400	35 ± 11	<0.001
Mitral valve E-wave, m/s	627	0.7 ± 0.3	208	0.8 ± 0.3	419	0.7 ± 0.2	<0.001
Medial E/e’ ratio	504	9 ± 6	161	11 ± 7	343	8 ± 5	<0.001
≥Moderate mitral regurgitation	482	21 (4.0)	178	15 (8.0)	304	6 (2.0)	0.001
LV end-diastolic diameter, mm	670	43 ± 6	232	44 ± 7	438	42 ± 6	0.010
LV end-systolic diameter, mm	660	28 ± 6	228	29 ± 6	432	28 ± 5	0.002
LV ejection fraction, %	676	58 ± 8	233	57 ± 9	443	59 ± 7	0.004

Values are N, n (%), or mean ± SD.

AA = atrial arrhythmia; CIED = cardiac implantable electronic device; LA = left atrial; LV = left ventricle; RA = right atrial; RV = right ventricle; TAPSE = tricuspid annular plane systolic excursion; TR = tricuspid regurgitation.

a Strain was modeled as absolute values.

In the management of the initial episodes of AA, 101 (43%) received AAD (Class I AAD 38 [16%] and Class III AAD 63 [27%]), and 74 (31%) underwent direct current cardioversion for episode termination. At the time of baseline evaluation, 72 patients (31%) were on AAD (Class I AAD 29 [12%] and Class III AAD 43 [18%]), 23 (10%) had percutaneous catheter ablation, 5 (2%) had RA Maze, and 1 (0.4%) had left atrial Maze.

**Figure 1:**
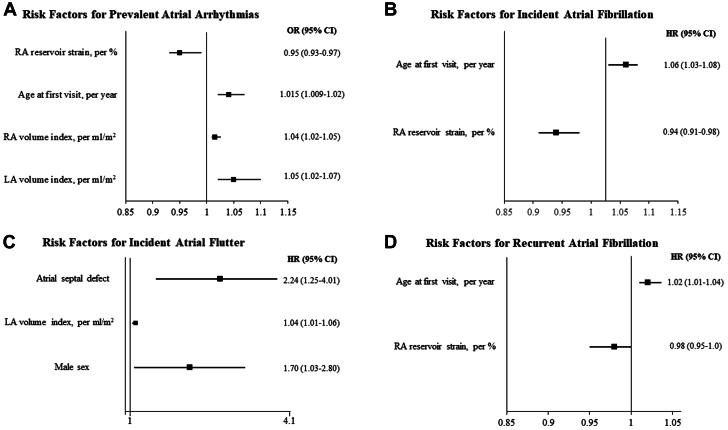
**Independent Risk Factors for the Development of Atrial Arrhythmias** Multivariable regression models depicting risk factors for prevalent atrial arrhythmias at baseline **(A)**, incident atrial fibrillation **(B)**, incident atrial flutter/tachycardia **(C)**, and recurrent atrial fibrillation **(D)**. Note that data for incident atrial flutter are presented in logarithmic scale for better visualization. HR = hazard ratio; LA = left atrial; OR = odds ratio; RA = right atrial.

The univariable and multivariable models for the risk factors associated with prevalent AA are shown in Supplemental Table 1 and Table 2, respectively. RA reservoir strain and volume index, older age at first visit, and left atrial volume index were independently associated with prevalent AA (Figure 1A).

**Table 2:** Risk Factors Associated With Atrial Arrhythmias at Baseline

	Multivariable Analysis	
	OR (95% CI)	*P* Value
RA reservoir strain^a^, per %	0.95 (0.93-0.97)	<0.001
RA volume index, per mL/m^2^	1.015 (1.009-1.02)	<0.001
Age at first visit, per y	1.04 (1.02-1.05)	<0.001
LA volume index, per mL/m^2^	1.05 (1.02-1.07)	<0.001

LA = left atrial; OR = odds ratio; RA = right atrial.

a Strain was modeled as absolute values.

### Incident AA

**Figure 2:**
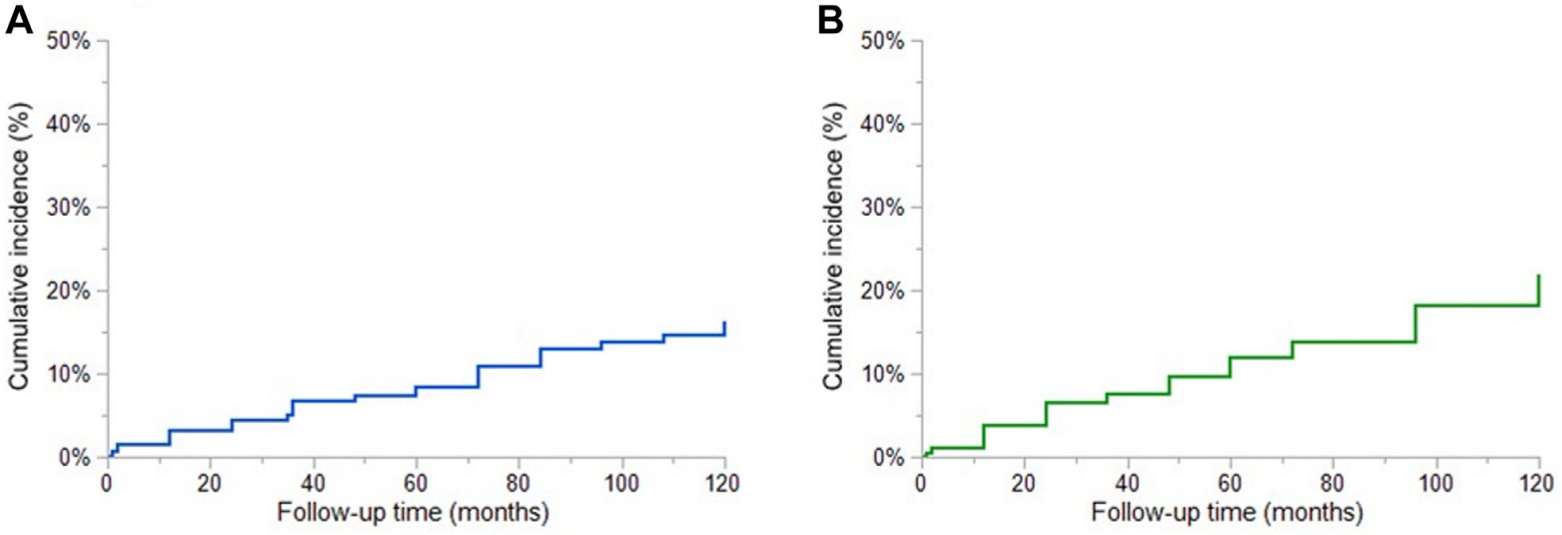
**Cumulative Incidence of New Atrial Arrhythmias** **(A)** Cumulative incidence of atrial fibrillation and **(B)** of atrial flutter calculated with the Kaplan-Meier method. The cumulative incidences of atrial fibrillation were 8% and 16% at 5 and 10 years, respectively. The cumulative incidences of atrial flutter were 12% and 22% at 5 and 10 years, respectively. Time 0 for assessing incident atrial arrhythmias was the baseline encounter.

The 447 patients without AA at baseline were followed for 24 months (1-108), and during this period, 44 (10%) had incident AF. The cumulative incidences of AF were 8% and 16% at 5 and 10 years, respectively (Figure 2A). The univariable and multivariable models for the risk factors associated with incident AF are shown in Supplemental Table 2 and Table 3, respectively. Older age at first visit and RA reservoir strain were independently associated with incident AF (Figure 1B).

**Table 3:** Multivariable Cox Model of Risk Factors Associated With Incident Atrial Fibrillation

	Multivariable Analysis	
	HR (95% CI)	*P* Value
Age at first visit, per y	1.06 (1.03-1.08)	<0.001
RA reservoir strain^a^, per %	0.94 (0.91-0.98)	<0.001

RA = right atrial.

a Strain was modeled as absolute values.

Sixty-seven (15%) of the 447 patients without prior history of AA developed incident AFL during follow-up. The cumulative incidences of AFL were 12% and 22% at 5 and 10 years, respectively (Figure 2B). The univariable and multivariable models for the risk factors associated with incident AFL are shown in Supplemental Table 3 and Table 4, respectively. Atrial septal defect, left atrial volume index, and male sex were independently associated with incident AFL (Figure 1C).

**Table 4:** Multivariable Cox Model of Risk Factors Associated With Incident Atrial Flutter/Tachycardia

	Multivariable Analysis	
	HR (95% CI)	*P* Value
Atrial septal defect	2.24 (1.25-4.01)	0.004
LA volume index, per mL/m^2^	1.04 (1.01-1.06)	0.012
Male	1.70 (1.03-2.80)	0.043

LA = left atrial.

### Recurrent AA

In addition to the 235 patients with prevalent AA at baseline, 87 patients developed incident AA during follow-up. Altogether, 322 patients (47%) had at least 1 episode of AA at baseline or during follow-up. These 322 patients received the following antiarrhythmic therapies after their initial episode of AA: AAD 58 (18%), catheter ablation 94 (29%), antiarrhythmic surgery (121 patients [38%] RA Maze, 1 [0.3%] left atrial Maze, and 25 [8%] biatrial Maze). Table 5 shows the details of the received antiarrhythmic therapies.

**Table 5:** Rhythm Control Therapy at Last Follow-Up

	N	n (%)
Episode management	322	
Class I antiarrhythmics^a^		26 (8.0)
Class III antiarrhythmics^b^		120 (37.0)
Direct current cardioversion		122 (38.0)
Antiarrhythmic drugs at last follow-up	322	
Class I antiarrhythmics^a^		7 (2.0)
Class III antiarrhythmics^b^		51 (16.0)
Percutaneous catheter ablation	322	
Atrial fibrillation		9 (3.0)
Intra-atrial re-entrant tachycardia		
Typical atrial flutter		67 (21.0)
Atypical right atrial flutter		45 (14.0)
Atypical left atrial flutter		1 (0.3)
Focal atrial tachycardia		14 (4.0)
Antiarrhythmic surgery	322	
Right atrial Maze		121 (38.0)
Left atrial Maze		1 (0.3)
Biatrial Maze		25 (8.0)

’N’ signifies number of patients with available data and ‘n’ signifies number of patients with positive results for each variable.

a Quinidine, procainamide, flecainide, propafenone.

b Sotalol, dofetilide, amiodarone.

**Figure 3:**
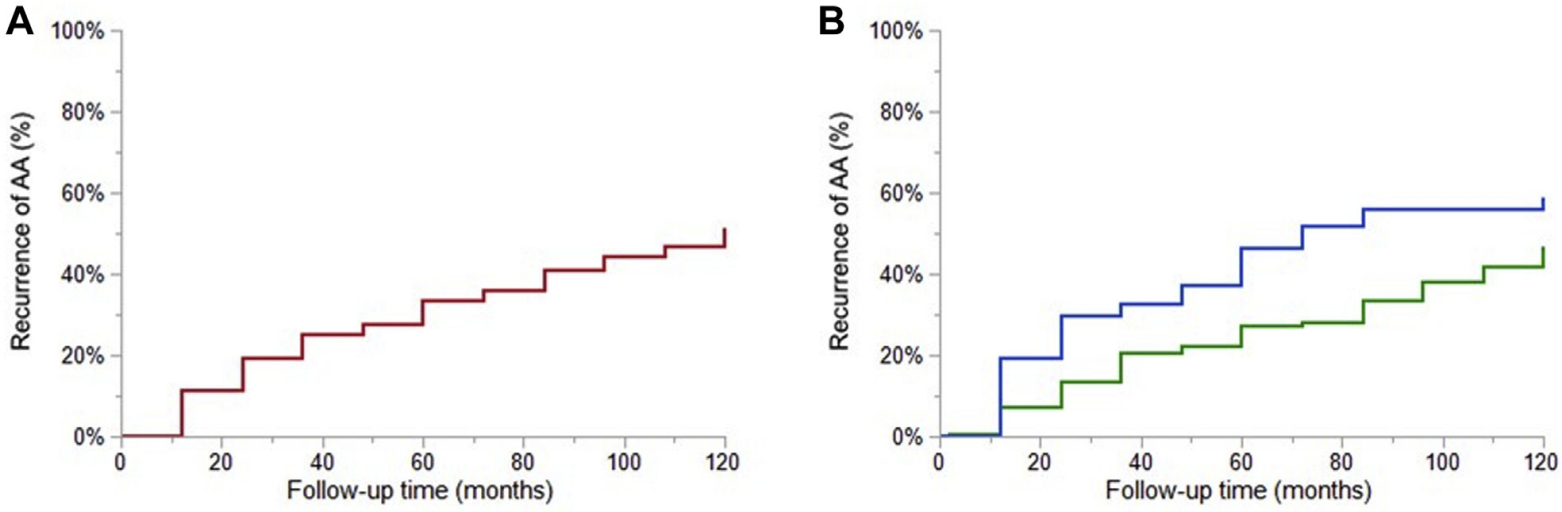
**Cumulative Incidence of Recurrent Atrial Arrhythmias** Calculation was performed with the Kaplan-Meier method. **(A)** Cumulative incidence of recurrent atrial arrhythmias was 34% and 51% at 5- and 10-year follow-up, respectively. **(B)** The 5-year cumulative incidence of recurrent atrial arrhythmias was 46% in patients with atrial fibrillation at baseline **(blue)** vs 27% in those with absence of atrial fibrillation at baseline **(green)**, *P* = 0.081. In patients with a history of AA, time 0 is at the first clinical encounter. In patients without a history of AA at the first clinical encounter, time 0 is at the time at first incident AA. AA = atrial arrhythmia.

Of these 322 patients, 129 (40%) developed recurrent AA (AF 63 [20%] and AFL 78 [24%]). The 5- and 10-year cumulative incidence of recurrent AA was 34% and 51%, respectively, and this did not differ significantly whether AF was present or absent at baseline (46% vs 27% at 5 years, respectively, *P* = 0.081) (Figures 3A and 3B). Because some patients had prior history of both AF and AFL before baseline, we performed the same analysis excluding those with prior history of both AA. The 5-year cumulative incidence of recurrent AA was similar regardless of whether the initial arrhythmia was AF or AFL (36% vs 33%, respectively, *P* = 0.854) (Supplemental Figure 1).

The univariable and multivariable models for the risk factors associated with recurrent AF are shown in Supplemental Table 4 and Table 6, respectively. Older age at baseline and RA reservoir strain were associated with recurrent AF, the latter with borderline statistical significance (Figure 1D). The univariable model for the risk factors associated with recurrent AFL is shown in Supplemental Table 5. Older age, prevalence of AF at baseline, hypertension, and catheter ablation were associated with recurrent AFL in the univariable model, but no variable remained significant after adjusting for the rest of covariates in the multivariable model.

**Table 6:** Multivariable Cox Model of Risk Factors Associated With Recurrent Atrial Fibrillation

	Multivariable Analysis	
	HR (95% CI)	*P* Value
Age at first visit, per y	1.02 (1.01-1.04)	<0.001
RA reservoir strain^a^, per %	0.98 (0.95-1.00)	0.054

RA = right atrial.

a Strain was modeled as absolute values.

## Discussion

We performed a comprehensive analysis of AA in a large cohort of adults with EA to assess arrhythmia burden and risk factors for recurrence. The main findings of the study are (Central Illustration) as follows. 1) AA was common at the time of initial presentation (prevalence of 34%), with AF occurring almost as frequently as AFL despite the relatively young age of the cohort; the risk factors associated with prevalent AA were older age, RA dilation and dysfunction, and left atrial dilation. 2) Among patients without AA at baseline, the 10-year cumulative incidence of AF and AFL were 16% and 22%, respectively, and the risk factors associated with incident AF and AFL were older age, male sex, atrial septal defect, left atrial dilation, and RA dysfunction. 3) Among patients with prior history of AA, the 5-year cumulative risk of arrhythmia recurrence was high (34%), and the risk did not differ significantly whether the initial AA was AF or AFL.

**Central Illustration:**
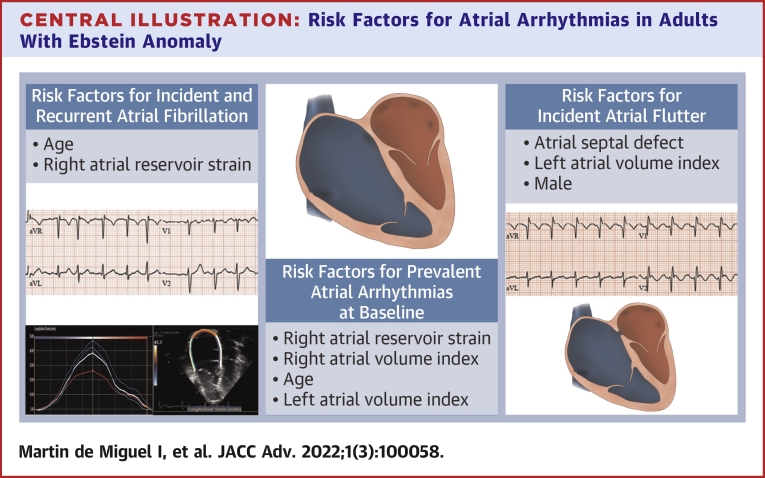
**Risk Factors for Atrial Arrhythmias in Adults With Ebstein Anomaly** This illustration depicts independent predictors for prevalent atrial arrhythmias at baseline, incidental atrial flutter and incidental and recurrent atrial fibrillation at last follow-up. Disease severity represented by right and left chambers’ size and function metrics (right atrial reservoir strain, left atrial volume index), older age at presentation, male sex, and concomitant congenital anomalies (atrial septal defect) were independently associated with higher risk of atrial arrhythmias.

AA is common in adults with congenital heart disease (CHD), and relative frequencies of AF and AFL vary depending on CHD complexity and the demographic characteristics of the cohort.^7^^,^^24^, ^25^, ^26^ Studies conducted in patients with EA have reported supraventricular arrhythmia prevalence ranging from 30% to 40%.^2^ However, they included both AA and paroxysmal supraventricular tachycardia (SVT). We chose to excluding SVT from our analyses because the pathophysiology, natural history, and outcomes in SVT vs AF and AFL differ significantly.^5^^,^^8^^,^^9^^,^^16^ While SVT arises from congenital anomalies involving accessory pathways and reentry circuits around the atrioventricular node, AA results from atrial remodeling and fibrosis.^8^^,^^9^^,^^27^^,^^28^ This is consistent with the relationship between atrial dilation and dysfunction (imaging markers of atrial remodeling and fibrosis), and the risk of AA observed in the present study. In our cohort, AF was very common and almost as prevalent as AFL, despite the relatively young age of our patients. Previously published data on distribution and age-related patterns of AA in CHD have reported AF onset at similar ages as in our cohort; however, these studies included solely patients with AA; hence, assessment of AF prevalence was not feasible, and they comprised heterogenous cohorts including complex CHD.^24^^,^^29^ Population-based studies of patients with CHD have shown a significantly lower AF prevalence (8.3%) at similar ages as our patients (42 years),^25^ whereas a prevalence similar to ours of 18% has been reported above the age of 60 years.^26^ We postulate that our significantly higher prevalence of AF at younger ages is driven by more significant abnormalities in the RA at an earlier age in EA than in other CHD. Indeed, these patients combine the intrinsic arrhythmic substrate from congenital conduction abnormalities, the altered right chamber physiology present since fetal life derived from their abnormal anatomy, and additional surgical manipulation, all of which results in a particularly high arrhythmic vulnerability.^8^^,^^9^

Our results show a 5-year recurrence rate of AA of 34%, which is higher than previously reported rates of 21% to 25% in patients with EA undergoing surgical ablation.^12^^,^^15^ However, differences in follow-up duration and study populations (as these studies solely included patients with AA undergoing Maze procedures) limit comparisons. Data from the precone repair era showed a 33% recurrence rate of AF/AFL in a significantly younger cohort.^30^ Finally, small studies analyzing specifically recurrence after catheter ablation observed a 5-year recurrence rate of ∼40%.^11^^,^^18^ We found predictors for AF recurrence, but not for AFL, and interestingly, recurrence rates did not differ significantly according to whether AF or AFL was the initial presenting AA. Most certainly, the progressive nature of tricuspid valve disease in EA perpetuates adverse atrial structural and electrical remodeling, fostering AF/AFL recurrence.^27^^,^^28^

Another important observation from the present study was that even patients without prior history of AA were at risk for incident AA and that assessment of atrial size and function can stratify those patients. Furthermore, atrial function can also identify patients at risk for recurrent AF. This could have potential clinical implications for risk stratification and could eventually be used to identify the most vulnerable patients that could be considered for intensive antiarrhythmic therapy to prevent arrhythmia recurrence. Specifically, left atrial volume index was associated with incident AFL and RA reservoir strain with incident and recurrent AF. Decreased reservoir strain reflects less atrial deformation during atrial filling, indicating adverse remodeling and fibrosis, and correlates with filling pressures,^31^ hence augmented arrhythmic risk, which persists despite tricuspid valve intervention. Left atrial volume index and RA strain might thus represent more sensitive diagnostic markers of atrial myopathy and worse hemodynamic status than other echocardiographic metrics, and our results suggest that they could be therefore used for the identification of patients at higher risk of AA development. Histologic analysis of a small cohort of adults with EA showed greater RA fibrosis in those with AA and good correlation between AA and noninvasive fibrosis measurement by P-wave dispersion; the latter increasing after surgery despite reduction in RA size and volume offloading, suggesting irreversibility of conduction disturbances.^32^ We observed that RA reservoir strain was associated with AF, whereas a metric related to the left atrium predicted incident AFL. These findings probably reflect diffuse atrial myopathy affecting both atria^11^^,^^32^; in addition, AF triggers outside the pulmonary veins, including RA and superior vena cava have been described in CHD.^7^^,^^27^

Clinical predictors of AA were male sex, older age, and atrial septal defects. Male sex was a risk factor for AFL incidence, in accordance with population-based data in CHD.^26^ The association between atrial septal defect and AA is well known, especially with late defect closure.^33^ Age was a predictor of AF, but not of AFL. Patients with EA developing AF may represent an older subgroup with more severe atrial fibrosis from longstanding volume and pressure overload. Indeed, age has been consistently associated with increased risk of AF in CHD,^25^^,^^29^ whereas AFL, conversely, has been related to prior cardiac operations, among others, because of surgical scars that provide substrate for macroreentry circuits.^7^^,^^24^ Although surgical interventions were common in our cohort and were associated with less AF incidence and recurrence and higher AFL incidence in univariable analysis, the association was no longer present after adjusting for other covariates. This may relate to other factors being more relevant, although the performance of Maze procedure at the time of surgery in a high proportion of patients compared with other CHD may have diminished the proarrhythmic effect of atriotomy incisions. In addition, the hemodynamic unloading by correction of tricuspid regurgitation through valve repair/replacement could further contribute to a reduction of arrhythmic risk, complicating the understanding of the role of tricuspid valve surgery in terms of AA development.

An important observation from this study was the lack of correlation between the different types of antiarrhythmic therapies and AA recurrence. One possible explanation is that all antiarrhythmic therapies are equally effective. Alternatively, there might have been a selection bias in the types of therapies offered to the patient because this is a nonrandomized study. A prospective study will be required to determine the optimal therapy in this population. It should be noted that long-term treatment with AAD was uncommon, probably because of the lack of efficacy and/or concern for proarrhythmic risk and other side effects,^7^^,^^34^ and catheter ablation was performed in less than one-third of the cohort, all of which could have impacted our results.

### Future directions and clinical implications

Our results reinforce previously published data of high AA burden in EA and expand current knowledge by providing simple echocardiographic metrics associated with higher AA risk. We therefore encourage clinicians and imagers to include atrial strain and volumetric quantification of atrial size in routine echocardiography practice, which, in conjunction with clinical evaluation, might help identify patients at particularly high arrhythmic risk. AA recurrences have significant clinical implications^34^^,^^35^ and remain the most common cause of hospitalization in adults with CHD, including EA.^1^^,^^2^^,^^36^ An important question remains as to whether these parameters may help guide antiarrhythmic therapy and ultimately improve patients’ outcome.

### Study Limitations

Our study has the inherent limitations of a retrospective design, and data come from a quaternary care center with extensive experience in managing EA, so referral bias is expected. We report AFL in conjunction with focal atrial tachycardia; however, the latter represented a minority of cases; therefore, the present results should be extrapolated to those presenting with atrial tachycardia with caution. Collinearity between older age at presentation and RA reservoir strain cannot be completely excluded; nevertheless, lower RA strain values have been reported in young patients with EA compared with healthy subjects,^23^ and correlation between this metric and exercise capacity and biventricular systolic function has been documented,^37^ suggesting high hemodynamic stress from tricuspid regurgitation and right ventricular systolic and diastolic dysfunction at early ages in this population. Furthermore, to the best of our knowledge, this is the largest cohort of EA reported to date with comprehensive analysis of AA.

## Conclusions

AA represents a significant cause of morbidity in EA with high recurrence rates, despite improvement in surgical techniques and trends toward earlier intervention. AF was highly prevalent and occurred almost as frequently as AFL, despite the relatively young age of the cohort. Disease severity represented by right and left chambers’ size and function metrics (RA reservoir strain and left atrial volume index) is associated with incident and recurrent AA, which can help identify patients at higher arrhythmic risk in clinical practice. Further studies are required to develop interventions targeted at the risk factors associated with AA in this population.

PERSPECTIVES **COMPETENCY IN MEDICAL KNOWLEDGE:** Despite the improvement of surgical techniques and earlier intervention, adults with EA are at significant risk for incident and recurrent AA. We provide simple echocardiographic metrics associated with higher AA risk, which might be used, in conjunction with clinical evaluation, to improve patients’ risk stratification and guide therapy.

**TRANSLATIONAL OUTLOOK:** Further studies are warranted to develop interventions targeted at the risk factors associated with AA and to assess whether these independent predictors of higher arrhythmic risk may help guide antiarrhythmic therapy and ultimately improve clinical outcomes.

## Funding support and author disclosures

Dr Egbe is supported by National Heart, Lung, and Blood Institute (NHLBI) grants (R01 HL158517 and R01 160761). The MACHD Registry is supported by the Al-Bahar research grant. All other authors have reported that they have no relationships relevant to the contents of this paper to disclose.
