# Supplementary material for: Risk Factors for Atrial Arrhythmias in Adults With Ebstein Anomaly
Source: JACC Adv. 2022 Aug 26;1(3):100058. doi: 10.1016/j.jacadv.2022.100058 (PMC11198643; doi:10.1016/j.jacadv.2022.100058)
Supplement: Supplemental Tables 1–5 and Figure 1 [file mmc1.docx]

**Supplemental Table 1: Risk Factors Associated with Atrial Arrhythmias at Baseline. Univariable and Multivariable Model**

|  | **Univariable analysis** | | **Multivariable analysis** | |
| --- | --- | --- | --- | --- |
| **Variables** | **OR (95%CI)** | **p** | **OR (95%CI)** | **p** |
| Age at first visit, per year | 1.05 (1.03-1.06) | <0.0001 | 1.04 (1.02-1.05) | <0.0001 |
| Male sex | 1.61 (1.17-2.22) | 0.004 |  |  |
| Hypertension | 1.71 (1.12-2.61) | 0.01 |  |  |
| Dyslipidemia |  | --- |  |  |
| Diabetes mellitus |  | --- |  |  |
| Obesity |  | --- |  |  |
| Previous stroke |  | --- |  |  |
| Creatinine, mg/dl | 2.10 (1.04-4.23) | 0.04 |  |  |
| Atrial septal defect | 1.35 (0.98-1.85) | 0.07 |  |  |
| Ventricular septal defect |  | --- |  |  |
| Pulmonary stenosis |  | --- |  |  |
| Prior cardiac surgery | 1.88 (1.32-2.67) | 0.0005 |  |  |
| RA volume index, per ml/m^2^ | 1.015 (1.010-1.02) | <0.0001 | 1.015 (1.009-1.02) | <0.0001 |
| RA reservoir strain*, per % | 0.93 (0.91-0.94) | <0.0001 | 0.95 (0.93-0.97) | <0.0001 |
| RA pressure, per mmHg | 1.11 (1.07-1.15) | <0.0001 |  |  |
| ≥ moderate tricuspid regurgitation# | 0.67 (0.47-0.96) | 0.03 |  |  |
| RV systolic pressure, per mmHg |  | --- |  |  |
| RV end-diastolic area, per cm^2^ | 1.02 (1.01-1.03) | 0.002 |  |  |
| RV global strain*, per % | 0.91 (0.88-0.94) | <0.0001 |  |  |
| LA volume index, per ml/m^2^ | 1.06 (1.05-1.08) | <0.0001 | 1.05 (1.02-1.07) | <0.0001 |
| LA reservoir strain*, per % | 0.94 (0.93-0.96) | <0.0001 |  |  |
| Medial E/e’ ratio, per unit | 1.08 (1.04-1.12) | <0.0001 |  |  |
| ≥ moderate mitral regurgitation# | 4.57 (1.74-12.01) | 0.001 |  |  |
| LV end-diastolic diameter, per mm | 1.03 (1.01-1.06) | 0.01 |  |  |
| LV ejection fraction, per % | 0.97 (0.95-0.99) | 0.004 |  |  |

CI: confidence interval; LA: left atrial; LV: left ventricle; OR: odds ratio; RA: right atrial; RV: right ventricle.

*Strain was modeled as absolute values.

#By echocardiographic assessment.

--- denoted variables with p>0.1; a threshold of p≤0.1 in the univariable analysis was required to enter the multivariable model.

**Supplemental Table 2: Multivariable Cox Model of Risk Factors Associated with Incident Atrial Fibrillation. Univariable and Multivariable Model**

|  | **Univariable analysis** | | **Multivariable analysis** | |
| --- | --- | --- | --- | --- |
| **Variables** | **HR (95%CI)** | **p** | **HR (95%CI)** | **p** |
| Age at first visit, per year | 1.05 (1.02-1.07) | <0.0001 | 1.06 (1.03-1.08) | <0.0001 |
| Male sex | 1.18 (0.64-2.18) | 0.6 |  |  |
| Hypertension |  | --- |  |  |
| Dyslipidemia |  | --- |  |  |
| Diabetes mellitus |  | --- |  |  |
| Obesity |  | --- |  |  |
| Previous stroke |  | --- |  |  |
| Creatinine, mg/dl | 3.34 (1.21-7.18) | 0.007 |  |  |
| Atrial septal defect | 1.88 (0.96-3.67) | 0.07 |  |  |
| Ventricular septal defect |  | --- |  |  |
| Pulmonary stenosis |  | --- |  |  |
| Prior cardiac surgery | 0.36 (0.14-0.91) | 0.03 |  |  |
| Antiarrhythmic drug at baseline |  | --- |  |  |
| Prior electrophysiological study |  | --- |  |  |
| RA volume index, per ml/m^2^ |  | --- |  |  |
| RA reservoir strain*, per % | 0.95 (0.92-0.98) | 0.006 | 0.94 (0.91-0.98) | 0.0008 |
| RA pressure, per mmHg |  | --- |  |  |
| ≥ moderate tricuspid regurgitation# |  | --- |  |  |
| RV systolic pressure, per mmHg |  | --- |  |  |
| RV end-diastolic area, per cm^2^ |  | --- |  |  |
| RV global strain*, per % | 0.95 (0.90-1.005) | 0.007 |  |  |
| LA volume index, per ml/m^2^ | 1.04 (1.003-1.06) | 0.02 |  |  |
| LA reservoir strain*, per % | 0.95 (0.92-0.98) | 0.004 |  |  |
| Medial E/e’ ratio, per unit |  | --- |  |  |
| ≥ moderate mitral regurgitation# |  | --- |  |  |
| LV end-diastolic diameter, per mm |  | --- |  |  |
| LV ejection fraction, per % | 0.96 (0.93-1.002) | 0.03 |  |  |

CI: confidence interval; HR: hazard ratio; LA: left atrial; LV: left ventricle; RA: right atrial; RV: right ventricle.

*Strain was modeled as absolute values.

#By echocardiographic assessment.

--- denoted variables with p>0.1; a threshold of p≤0.1 in the univariable analysis was required to enter the multivariable model.

**Supplemental Table 3: Multivariable Cox Model of Risk Factors Associated with Incident Atrial Flutter/** **Tachycardia. Univariable and Multivariable Model**

|  | **Univariable analysis** | | **Multivariable analysis** | |
| --- | --- | --- | --- | --- |
| **Variables** | **HR (95%CI)** | **p** | **HR (95%CI)** | **p** |
| Age at first visit, per year | 1.01 (0.99-1.03) | 0.2 |  |  |
| Male sex | 1.64 (1.008-2.67) | 0.05 | 1.70 (1.03-2.80) | 0.043 |
| Hypertension |  | --- |  |  |
| Dyslipidemia |  | --- |  |  |
| Diabetes mellitus |  | --- |  |  |
| Obesity |  | --- |  |  |
| Previous stroke |  | --- |  |  |
| Creatinine, mg/dl |  | --- |  |  |
| Atrial septal defect | 1.98 (1.15-3.42) | 0.01 | 2.24 (1.25-4.01) | 0.004 |
| Ventricular septal defect |  | --- |  |  |
| Pulmonary stenosis |  | --- |  |  |
| Prior cardiac surgery | 1.55 (0.92-2.60) | 0.1 |  |  |
| Antiarrhythmic drug at baseline |  | --- |  |  |
| Prior electrophysiological study |  | --- |  |  |
| RA volume index, per ml/m^2^ |  | --- |  |  |
| RA reservoir strain*, per % |  | --- |  |  |
| RA pressure, per mmHg |  | --- |  |  |
| ≥ moderate tricuspid regurgitation# |  | --- |  |  |
| RV systolic pressure, per mmHg |  | --- |  |  |
| RV end-diastolic area, per cm^2^ |  | --- |  |  |
| RV global strain*, per % | 0.97 (0.93-1.01) | 0.1 |  |  |
| LA volume index, per ml/m^2^ | 1.03 (1.01-1.06) | 0.008 | 1.04 (1.01-1.06) | 0.012 |
| LA reservoir strain*, per % | 0.97 (0.95-0.99) | 0.02 |  |  |
| Medial E/e’ ratio, per unit |  | --- |  |  |
| ≥ moderate mitral regurgitation# |  | --- |  |  |
| LV end-diastolic diameter, per mm |  | --- |  |  |
| LV ejection fraction, per % |  | --- |  |  |

CI: confidence interval; HR: hazard ratio; LA: left atrial; LV: left ventricle; RA: right atrial; RV: right ventricle.

*Strain was modeled as absolute values.

#By echocardiographic assessment.

--- denoted variables with p>0.1; a threshold of p≤0.1 in the univariable analysis was required to enter the multivariable model.

**Supplemental Table 4: Multivariable Cox Model of Risk Factors Associated with Recurrent Atrial Fibrillation. Univariable and Multivariable Model**

|  | **Univariable analysis** | | **Multivariable analysis** | |
| --- | --- | --- | --- | --- |
| **Variables** | **HR (95%CI)** | **p** | **HR (95%CI)** | **p** |
| Age at first visit, per year | 1.03 (1.01-1.05) | 0.0003 | 1.02 (1.01-1.04) | 0.005 |
| Male sex | 1.02 (0.61-1.68) | 0.95 |  |  |
| Atrial flutter/tachycardia at baseline |  | --- |  |  |
| Hypertension |  | --- |  |  |
| Dyslipidemia |  | --- |  |  |
| Diabetes mellitus |  | --- |  |  |
| Obesity |  | --- |  |  |
| Previous stroke |  | --- |  |  |
| Creatinine, mg/dl |  | --- |  |  |
| Atrial septal defect |  | --- |  |  |
| Ventricular septal defect |  | --- |  |  |
| Pulmonary stenosis |  | --- |  |  |
| Prior cardiac surgery | 0.49 (0.26-0.90) | 0.02 |  |  |
| Cardiac surgery* |  | --- |  |  |
| Antiarrhythmic drug at baseline |  | --- |  |  |
| Prior percutaneous catheter ablation |  | --- |  |  |
| Percutaneous catheter ablation* | 1.62 (0.98-2.69) | 0.06 |  |  |
| Prior RA / LA surgical maze |  | --- |  |  |
| RA / LA surgical maze* |  | --- |  |  |
| RA volume index, per ml/m^2^ |  | --- |  |  |
| RA reservoir strain#, per % | 0.97 (0.95-0.99) | 0.05 | 0.98 (0.95-1.0) | 0.054 |
| RA pressure, per mmHg |  | --- |  |  |
| ≥ moderate tricuspid regurgitation† |  | --- |  |  |
| RV systolic pressure, per mmHg |  | --- |  |  |
| RV end-diastolic area, per cm^2^ |  | --- |  |  |
| RV global strain#, per % |  | --- |  |  |
| LA volume index, per ml/m^2^ |  | --- |  |  |
| LA reservoir strain#, per % | 0.98 (0.95-1.003) | 0.09 |  |  |
| Medial E/e’ ratio, per unit |  | --- |  |  |
| ≥ moderate mitral regurgitation† |  | --- |  |  |
| LV end-diastolic diameter, per mm |  | --- |  |  |
| LV ejection fraction, per % |  | --- |  |  |

CI: confidence interval; HR: hazard ratio; LA: left atrial; LV: left ventricle; RA: right atrial; RV: right ventricle.

*At any time during the study period.

#Strain was modeled as absolute values.

†By echocardiographic assessment.

--- denoted variables with p>0.1; a threshold of p≤0.1 in the univariable analysis was required to enter the multivariable model.

**Supplemental Table 5: Multivariable Cox Model of Risk Factors Associated with Recurrent Atrial Flutter/** **Tachycardia. Univariable and Multivariable Model**

|  | **Univariable analysis** | | **Multivariable analysis** | |
| --- | --- | --- | --- | --- |
| **Variables** | **HR (95%CI)** | **p** | **HR (95%CI)** | **p** |
| Age at first visit, per year | 0.99 (0.97-1.002) | 0.08 |  |  |
| Male sex | 0.96 (0.61-1.50) | 0.8 |  |  |
| Atrial fibrillation at baseline | 0.60 (0.35-1.02) | 0.05 |  |  |
| Hypertension | 0.48 (0.21-1.10) | 0.08 |  |  |
| Dyslipidemia |  | --- |  |  |
| Diabetes mellitus |  | --- |  |  |
| Obesity |  | --- |  |  |
| Previous stroke |  | --- |  |  |
| Creatinine, mg/dl |  | --- |  |  |
| Atrial septal defect |  | --- |  |  |
| Ventricular septal defect |  | --- |  |  |
| Pulmonary stenosis |  | --- |  |  |
| Prior cardiac surgery |  | --- |  |  |
| Cardiac surgery* |  | --- |  |  |
| Antiarrhythmic drug at baseline |  | --- |  |  |
| Prior percutaneous catheter ablation |  | --- |  |  |
| Percutaneous catheter ablation* | 3.52 (2.24-5.52) | <0.0001 |  |  |
| Prior RA / LA surgical maze |  | --- |  |  |
| RA / LA surgical maze* |  | --- |  |  |
| RA volume index, per ml/m^2^ |  | --- |  |  |
| RA reservoir strain#, per % |  | --- |  |  |
| RA pressure, per mmHg |  | --- |  |  |
| ≥ moderate tricuspid regurgitation† |  | --- |  |  |
| RV systolic pressure, per mmHg |  | --- |  |  |
| RV end-diastolic area, per cm^2^ |  | --- |  |  |
| RV global strain#, per % |  | --- |  |  |
| LA volume index, per ml/m^2^ |  | --- |  |  |
| LA reservoir strain#, per % |  | --- |  |  |
| Medial E/e’ ratio, per unit |  | --- |  |  |
| ≥ moderate mitral regurgitation† |  | --- |  |  |
| LV end-diastolic diameter, per mm |  | --- |  |  |
| LV ejection fraction, per % |  | --- |  |  |

CI: confidence interval; HR: hazard ratio; LA: left atrial; LV: left ventricle; RA: right atrial; RV: right ventricle.

*At any time during the study period.

#Strain was modeled as absolute values.

†By echocardiographic assessment.

--- denoted variables with p>0.1; a threshold of p≤0.1 in the univariable analysis was required to enter the multivariable model.

**
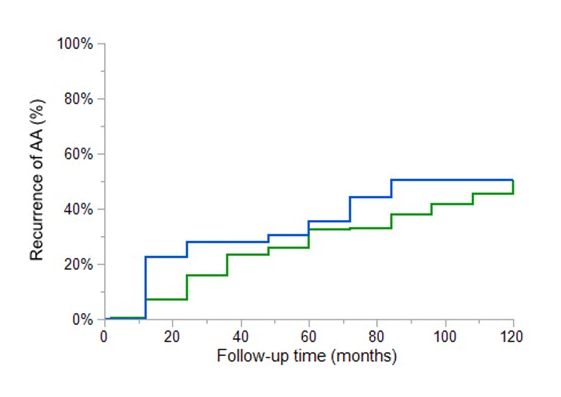
**

**Supplemental Figure 1. Cumulative incidence of recurrent atrial arrhythmias according to presence (blue) versus absence (green) of atrial fibrillation at baseline, excluding patients with both atrial fibrillation and atrial flutter/tachycardia at baseline**. The 5-year cumulative incidence of recurrent atrial arrhythmia was similar regardless of whether the initial arrhythmia was atrial fibrillation or atrial flutter (36% vs 33%, respectively, p=0.854). AA=atrial arrhythmia.
